# Pasteurella Pneumonia With Complicated Parapneumonic Effusion in a Pet Owner

**DOI:** 10.7759/cureus.79887

**Published:** 2025-03-01

**Authors:** Hajar El Amri, Soomal Rafique, Ali Zubairi

**Affiliations:** 1 Internal Medicine, Southern Illinois University School of Medicine, Springfield, USA; 2 Pulmonology, Southern Illinois University School of Medicine, Springfield, USA

**Keywords:** antibiotic therapy, complicated parapneumonic effusions, empyema, pasteurella multocida, pneumonia

## Abstract

*Pasteurella multocida* is a gram-negative coccobacillus commonly found in the oral flora of cats and dogs. While often associated with soft tissue infections, such as cellulitis and skin abscess following animal bites or scratches, empyema due to *P. multocida* is rare and primarily described in case reports. We present a case of a middle-aged woman with severe chronic obstructive pulmonary disease (COPD), diagnosed with empyema with a complicated course that required long-term antibiotic therapy. Her sputum cultures grew *P. multocida; *despite* *living with pet dogs and cats, she denied any recent bites or scratches. This case emphasizes the need for heightened awareness of *P. multocida* as a respiratory pathogen, particularly in patients with pet exposure and significant comorbidities. Early diagnosis and targeted treatment are crucial for managing this rare but serious infection effectively.

## Introduction

*Pasteurella multocida*, commonly associated with soft tissue infections following animal bites or scratches, can also cause severe respiratory infections in individuals with underlying pulmonary disease or immunocompromised states, even without a known bite or injury. Airborne contamination and chronic bacterial carriage in pet owners are documented mechanisms of exposure [1-3]. A review of 108 patients having respiratory infection with *P. multocida* reported 49 cases of pneumonia, 37 of tracheobronchitis, 25 of pleural empyema, and 3 of lung abscess [1]. These complications, which are associated with high mortality, are attributed to the pathogenicity of the bacterium [4,5]. Empyema due to *P. multocida* is, however, largely confined to case report literature [6]. We present a rare case of *P. multocida *pneumonia complicated by empyema in a patient with severe chronic obstructive pulmonary disease (COPD), alcoholic cirrhosis, and other significant comorbidities, despite no history of animal bites or scratches. This case underscores the importance of considering zoonotic infections in the appropriate clinical context and initiating prompt, targeted management.

## Case presentation

A 58-year-old woman with nonischemic cardiomyopathy, severe COPD, pulmonary hypertension, and alcoholic cirrhosis presented to the emergency room (ER) with a three-week history of fever, rigors, worsening dyspnea, and a cough productive of thick, purulent phlegm. She lived alone with her pet dogs and cats at home but denied any recent bites, scratches, or open wounds. Upon arrival to the ER, she was hypotensive and hypoxic, requiring 6 L of oxygen via nasal cannula. Examination revealed labored breathing with decreased breath sounds, particularly on the right side.

**Figure 1:**
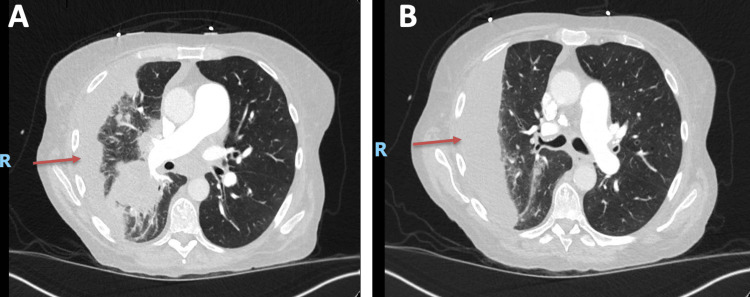
(A, B) Computed tomography of the chest with contrast on admission: consolidation involving the right middle and lower lobes and moderate-sized right partially loculated pleural effusion

The initial workup, as shown in Table 1, revealed severe leukocytosis, elevated procalcitonin and C-reactive protein levels, and mild hyponatremia. A CT angiography of the chest showed consolidation in the right middle and lower lobes with an associated right pleural effusion, as shown in Figure 1.

**Table 1:** Pertinent laboratory workup

Serum parameter	Results	Normal range
White blood cell count (K/cumm)	35	4-10
Procalcitonin (ng/mL)	5.54	<0.46
C-reactive protein (mg/L)	179	<5
Sodium (mmol/L)	130	136-145
Glucose (mg/dL)	205	70-110
Lactate dehydrogenase (IU/L)	125	140-271
Total protein (g/dL)	4.9	6.0-8.3

The patient was admitted to the intensive care unit for the management of septic shock with intravenous fluids and broad-spectrum antibiotics. Respiratory viral panel and atypical pneumonia workup (legionella urine antigen and mycoplasma antibodies) were negative. A diagnostic and therapeutic thoracentesis was performed, removing 530 mL of murky fluid. A pigtail catheter was placed for drainage, and intrapleural dornase alfa and tissue plasminogen activator (tPA) were administered for the loculated effusion. Pleural fluid analysis suggested empyema, with an elevated white blood cell count (neutrophilic predominance), a high lactate dehydrogenase, low glucose, a pleural fluid-to-serum lactate dehydrogenase (LDH) ratio of 15.5, a protein ratio of 0.6, and a gram stain showing few gram-positive cocci in pairs, which later showed no growth, as detailed in Table 2.

**Table 2:** Pleural fluid studies LDH: lactate dehydrogenase.

Parameter	Results	Normal range
White blood cell count (per cumm)	42,403	<1000
Neutrophils (% of white blood cell count)	92	<1
pH	7	7.60-7.64
Glucose (mg/dL)	<10	Similar to serum glucose
Protein (gm/dL)	<3	1-2
Lactate dehydrogenase (U/L)	1,948	<50% of serum LDH
Gram stain	Gram-positive cocci in pairs	Negative
Culture	No growth	No growth

However, the sputum culture initially showed gram-negative rods, which were later identified as *P. multocida *(sensitive to penicillin, ampicillin, amoxicillin, cefuroxime, cefpodoxime, doxycycline, levofloxacin, and trimethoprim-sulfamethoxazole)*. *Based on the sensitivities, cefepime was switched to ceftriaxone, while vancomycin and metronidazole were continued. Due to her comorbidities, surgical intervention with video-assisted thoracic surgery (VATS) was deferred in favor of medical management.

**Figure 2:**
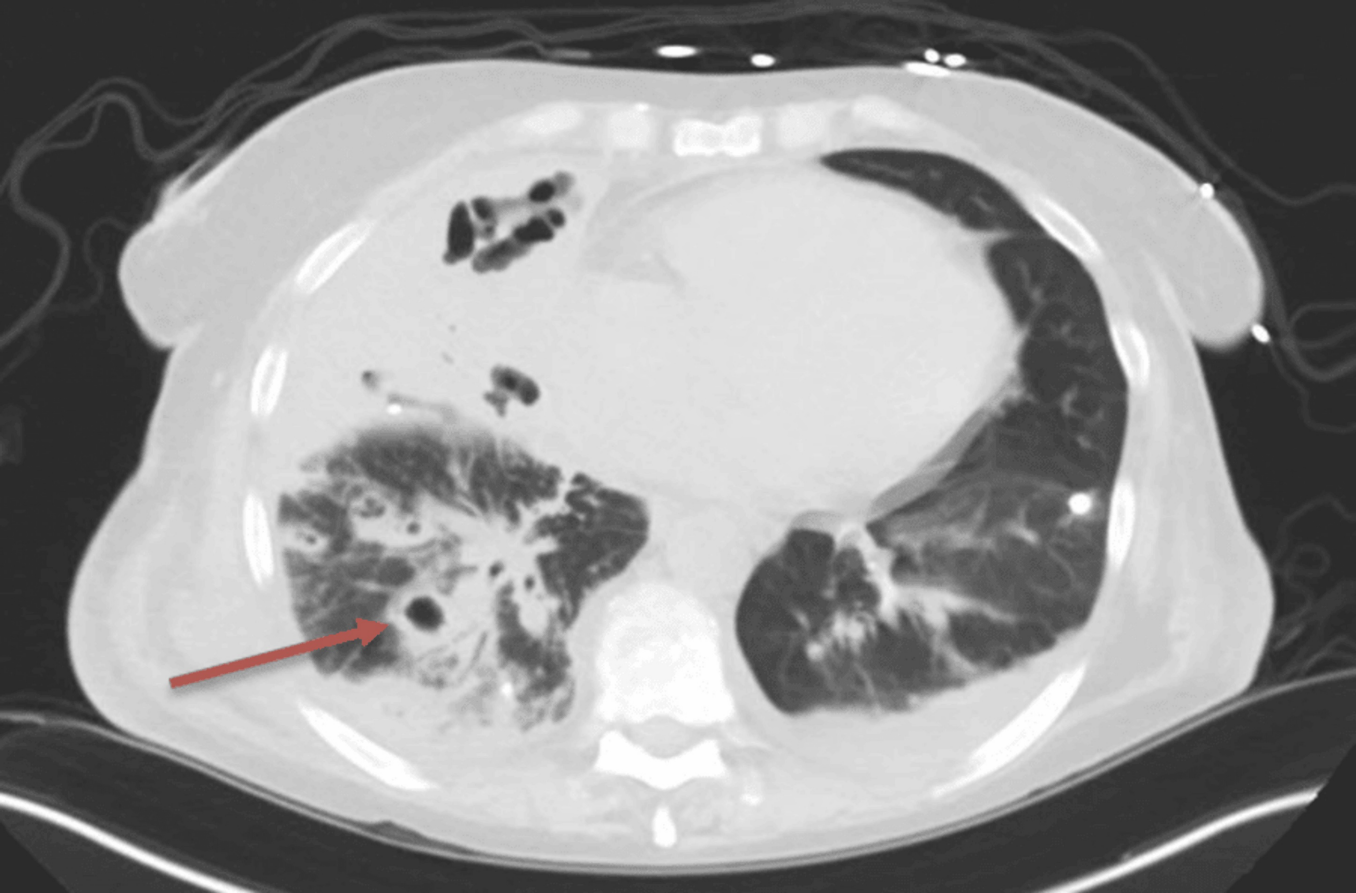
CT of the chest after pigtail chest tube removal: consolidative changes with cavitation in the right middle and lower lobes, along with scattered ground-glass and reticulonodular infiltrates bilaterally, and a resolved pleural effusion

A repeat CT of the chest, after chest tube removal, showed resolution of empyema and improvement in consolidation but revealed multiple cavitations concerning lung abscesses, as shown in Figure 2. Clinically, the patient improved with reduced oxygen requirements, hemodynamic stability, and resolution of her cough and dyspnea at rest. She was subsequently downgraded to a general floor and discharged on minimal oxygen with a tentative four-week course of antibiotic treatment and repeat CT with contrast in three weeks.

## Discussion

*P. multocida* is a nonmobile, gram-negative coccobacillus and a common commensal organism in the upper respiratory tracts of dogs and cats. In humans, it most frequently causes wound infections, such as cellulitis and skin abscesses, following animal bites or scratches. However, it can also lead to respiratory infections, the second most common site of infection. This is especially true in individuals with predisposing factors or comorbidities. In our case, the patient had severe COPD and alcoholic liver disease and owned multiple pets but did not recall any recent bites or scratches. There is documented evidence in the literature of airborne contamination and chronic bacterial carriage in pet owners as mechanisms of exposure [1-3].

*P. multocida* pneumonia commonly presents as lobar pneumonia, though it can also manifest as tracheobronchitis or, less frequently, lung abscesses. The prevalence of lung abscesses is not well documented in the literature. Empyema, although rare, is another possible complication. Respiratory infections carry a high mortality rate of up to 30% [4], primarily due to the combination of the patient’s underlying health conditions and the virulence of the organism itself.

Patients with compromised immune systems, such as those with cancer, diabetes, or liver disease, are at increased risk for severe infections. Those with preexisting lung conditions, including COPD or bronchiectasis, are particularly vulnerable. The pathogenicity of the bacterium is linked to its production of several virulence factors, such as cytotoxins that cause lung tissue damage, neuraminidase that facilitates bacterial spread and evasion of immune defenses, and a polysaccharide capsule and lipopolysaccharides that enhance resistance to complement-mediated killing and phagocytosis [5]. These mechanisms contribute to the rapid progression of the infection, leading to complications such as necrotizing pneumonia, sepsis, and organ failure.

In individuals with significant exposure to pets, the presence of* P. multocida* in sputum cultures may represent chronic colonization. However, in patients presenting with pneumonia, lung abscesses, or empyema, particularly with documented animal exposure, the isolation of *P. multocida* in sputum should be treated as evidence of true infection rather than colonization [7]. In the case of our patient, although empyema fluid cultures grew gram-positive cocci, the clinical presentation and the presence of risk factors supported *P. multocida* as the causative agent of the pneumonia and lung abscess.

Treatment of *P. multocida* infections generally involves beta-lactam antibiotics, with penicillins considered the first-line therapy [8]. Susceptibility testing is recommended, given the emergence of beta-lactamase-producing strains. Alternative antibiotics include doxycycline and fluoroquinolones. The recommended duration of therapy is 10-14 days for uncomplicated pneumonia, extending to 4-6 weeks in cases of lung abscess or empyema. Large or refractory abscesses may require percutaneous drainage to facilitate resolution [9].

## Conclusions

This case underscores the need to consider *P. multocida* a potential pathogen in pneumonia, particularly in individuals with close contact with domestic animals like cats and dogs. Pleural effusions may present as either exudative or transudative, with a wide variety of clinical presentations across patients. Infections of parapneumonic effusions can lead to empyema, which often results in prolonged hospital stays and increased mortality. Although *P. multocida* pneumonia is typically less severe and rarely leads to empyema (whether due to the same organism or others), early identification and targeted antibiotic therapy are essential for achieving favorable outcomes. This is especially important for vulnerable populations, such as the elderly or those with underlying health conditions.
